# Prognostic Value of Preoperative NLR and Vascular Reconstructive Technology in Patients With Pancreatic Cancer of Portal System Invasion: A Real World Study

**DOI:** 10.3389/fonc.2021.682928

**Published:** 2021-09-17

**Authors:** Lin Zhou, Jing Wang, Xin-xue Zhang, Shao-cheng Lyu, Li-chao Pan, Guo-sheng Du, Ren Lang, Qiang He

**Affiliations:** ^1^Department of Hepatobiliary and Pancreaticosplenic Surgery, Beijing ChaoYang Hospital, Capital Medical University, Beijing, China; ^2^Faculty of Hepato-Pancreato-Biliary Surgery, Chinese People’s Liberation Army (PLA) General Hospital, Beijing, China

**Keywords:** neutrophil-lymphocyte ratio, pancreatic ductal cell carcinoma, vascular invasion, curative resection, real world study

## Abstract

The purpose was aimed to establish a simple computational model to predict tumor prognosis by combining neutrophil to lymphocyte Ratio (NLR) and biomarkers of oncological characteristics in patients undergoing vascular reconstructive radical resection of PDAC. The enrolled patients was divided into high or low NLR group with the cutoff value determined by the receiver operator characteristic (ROC) curve. Different vascular anastomoses were selected according to the Chaoyang classification of PDAC. Survival rates were calculated using the Kaplan-Meier and evaluated with the log-rank test. Cox risk regression model was used to analyze the independent risk factors for prognostic survival. The optimal cut-off value of NRL was correlated with the differentiation, tumor size, TNM stage and distant metastasis of advanced PDAC. A curative resection with vascular reconstructive of advanced PDAC according to Chaoyang classification can obviously improve the survival benefits. Cox proportional hazards demonstrated higher evaluated NLR, incisal margin R1 and lymphatic metastasis were the independent risk predictor for prognosis with the HR > 2, meanwhile, age beyond 55, TNM stage of III-IV or Tumor size > 4cm were also the obvious independent risk predictor for prognosis with the HR ≤ 2. The advanced PADC patients marked of RS group (3 < RS ≤ 6) showed no more than 24 months of survival time according to RS model based on the six independent risk predictors. Vascular reconstruction in radical resection of advanced PDAC improved survival, higher elevated NLR (>2.90) was a negative predictor of DFS and OS in those patients accompanying portal system invasion.

## Introduction

Pancreatic ductal adenocarcinoma (PDAC) accounts for more than 90% (1) of all pancreatic cancer which is fifth most common cancers around the world (2). Although pancreatectomy is considered the only approach of curative treatment of PDAC, which provides a chance of cure and longer survival (3), but the prognosis is generally poor with a reported 5-year overall survival (OS) ranged from 10 to 30% postoperative (4–6). Once diagnosed, there are only about 10% of patients localized, meanwhile 29% of patients spread to regional lymph nodes with a relative 5-years low survival of 11.5%, compared with 34.3% for localized disease (7). In addition, about 80% of patients with PDAC experience a recurrence despite adjuvant therapy after a radical resection (8). Therefore radical resection, including thorough lymph node dissection, is an effective means to improve prognosis and survival.

Some studies have asserted that about 17-32% of patients with pancreatic cancer showed portal system including portal vein (PV), superior mesenteric vein (SMV) and splenic vein (SV) invasion once diagnosed (9). Among them, SMV and PV are the most vulnerable and frequent to invasion because of the proximity of these vessels to the uncinate process and pancreatic head (10). These patients may have a rather low median survival of 8 months compared with there were no vascular invasion (11). Radical resection of pancreatic cancer combined with complete vascular resection and reconstruction of the PV-SMV venous axis in these patients is a possible approach. The feasibility and advantages of this approach was proved, which may provide survival results comparable to those obtained with standard pancreatectomy without venous resection (12–14). That approach may improve the worse survival benefit with a OS of 18.2 months when only palliative treatment was given (15). Although vascular invasion as a prognostic factor was carried out in several studies which mainly focus on whether there is an association between vascular invasion and poor prognosis, the types of vascular invasion, classification (location, depth and circumference) and anastomotic techniques of vascular reconstruction on the prognosis is not clear.

Except the radical excision, early diagnosis is of great significance for the prognosis of pancreatic cancer patients. Carbohydrate antigen 19-9 (CA19-9) which is the only authenticated marker for clinical application, lacks the specificity required for a differential diagnosis (16). Searching for novel biomarkers to detect and diagnose PDAC earlier maybe another approach to improve the poor prognosis. Literatures of inflammatory indices and immunologic ratios, including ratios comprised of intratumoral or circulating neutrophils, platelets, lymphocytes, and monocyte counts, have been proposed to be prognostic biomarkers for a wide range of malignancies (17, 18). There has studies showed that neutrophil to lymphocyte ratio (NLR), not platelet to lymphocyte (PLR), is predictive on survival benefits after resection of early-stage PDAC (19, 20). The prognostic value of lymphocyte to monocyte ratio (LMR) levels for PDAC patients remains to be determined (19, 20). The relationship between NLR and prognosis of advanced PDAC after resection with vascular reconstruction remains unclear. Meanwhile, there exist few studies on the prediction of inflammatory markers, biomarkers of tumor characteristics and surgical techniques for OS and DFS in advanced PDAC patients.

This presented paper was aimed to explore the effect of NLR, tumor marker such as CA19-9, vascular reconstruction methods, lymphatic metastasis and other surgical and pathologically related factors on the long-term prognosis of PDAC with portal system invasion. Therefore, establishing a predictive model based on the risk factor of Cox regression analysis to predict OS and disease-free survival (DFS) after radical resection with vascular reconstruction of advanced PDAC is necessary and promising.

## Materials and Methods

### Patient Selection and Operative Techniques

#### Patient Selection

At the present study, we enrolled 241 patients who were diagnosed with pancreatic carcinoma from January 2011 to December 2019 and performed radical excision with strict criteria as follows. This study was approved by the Ethical Committee of Beijing Chao-Yang Hospital. All patients provided full written informed consent, which was obtained in accordance with the Declaration of Helsinki of the World Medical Association (Ethics approval and consent to participate: No.2020-D.-309-2). The authors are accountable for all aspects of the work in ensuring that questions related to the accuracy or integrity of any part of the work are appropriately investigated and resolved.

**Included criteria:** (1) Preoperative image indicated pancreatic malignancy. (2) Aged 20 to 85 years old. (3) En bloc resection of tumor during operation. (4) Postoperative pathology confirmed pancreatic ductal adenocarcinoma. (5) The mode of operation and treatment strategy obtained the informed consent of patients and their families.

**Excluded criteria:** (1) Unresectable condition or metastasis found during surgery. (2) Surgical rule violation. (3) Pathologic diagnosis other than conventional ductal adenocarcinoma. (4) Postoperative follow-up data were incomplete or lost to follow-up.

#### Operative Detections

Preoperative tumor evaluation was done by diagnostic imaging methods, including abdominal ultrasonography, computed tomography (CT) including lung and abdominal or abdominal magnetic resonance imaging (MRI). One should take a Positron Emission Tomography-Computed Tomography (PET-CT) or bone scan if distant metastasis is suspected. The laboratory measurement including liver function, tumor marker, hepatitis index, blood routine examination and thromboxane function.

#### Group and Operation

The patient compliance with the study criteria was admitted into the group. All the patients was divided into high NLR group and low NLR group which criteria for grouping as determined by ROC curves for healthy people and all the patients.

At present, there existed no uniform clinical standard for the classification of vascular invasion in pancreatic cancer. The most commonly used clinical standard for vascular invasion is the Loyer classification and Shibata typing (21, 22). However, all of the above classifications have certain limitations. On the one hand, it is impossible to assess the site and scope of tumor invasion to portal vein system, on the other hand, it has no guiding value for the resection and reconstruction of the invaded portal vein system. In recent years, our center has carried out a beneficial attempt to optimize the above vascular invasion typing criteria in patients treated with radical surgery and proposed a new typing system named Chaoyang classification (23). There are four types: (I) Portal and/or superior mesenteric vein invasions of less than 1/4 circumference. In this type of patients, the lateral wall of the vein can be blocked without blocking the blood flow into the liver. The affected side wall can be partially excised and the vein can be sutured directly. After suturing, the vein can be guaranteed to have no obvious stenosis. (II) Portal vein and/or superior mesenteric vein were invaded to a range greater than 1/4 circumference, or the vein was clearly narrowed and occluded, without involving the splenic vein junction. In this type of patients, segmental resection of the involved vein is recommended, and end-to-end anastomosis or allograft or artificial vascular reconstruction is selected according to the tension of the upper and lower edges. (III) The tumor invaded the confluence of portal vein, splenic vein and superior mesenteric vein. In this type of patients, partial splenic vein resection can be performed in conjunction with the confluence part, and splenic vein reconstruction can be completed by using foreign blood vessels with branches. (IV) The tumor invaded a wide area, the portal vein, splenic vein and superior mesenteric vein are involved in the upper part, and the branch of superior mesenteric vein in the lower part is involved. In this type of patients, arterial approach is recommended to complete tumor dissociation and then resection of invaded vessels, for reconstruction, it is recommended that the superior mesenteric vein branch be shaped into an opening first, and then Allogeneic blood vessels with branches or other substitutes should be used to complete the reconstruction. Different methods of vascular resection and reconstruction are adopted according to the specific form of venous invasion. The technique of vascular reconstruction and the type of pancreatic, biliary, and enteric anastomoses depended on operating surgeon’s choice.

According to the Chaoyang classification, about half of the patients included in this study are advanced PDAC with portal system invasion, with the standard of Chaoyang classification, we performed radical resection on the advanced pancreatic cancer, combined with vascular resection, reconstruction or allogeneic vascular replacement and lymph node dissection to meet the standard of R0 resection. Therefore, on the basis of NLR grouping, we used the operation mode, resection and reconstruction of invasive vessels in Chaoyang classification and the degree of tumor pathological differentiation (poorly differentiation, poorly-moderately differentiation, moderately differentiation and moderately-highly differentiation group) as subgroup criteria.

The follow up began when diagnosed and was in hospital, with whole data and records. The overall survival (OS) and disease-free survival (DFS) was the main index in measurement the survival benefits.

### Sample Detection and Hematoxylin-Eosin Stain

The pancreatic and vascular specimens were obtained once the tumor excision from the patients, and fixed with 10% formaldehyde solution. The 10% formalin fixed tissues embedded in paraffin, then microtome section with 5μm, heated at 60°C on slides warmer for 30 min, undergo the steps of dewaxing, benzene removal, hematoxylin and eosin staining, then dehydration and fixation.

### Statistical Analysis

Pathological results images were collected under optical microscopy for 40X, 100X and 200X visual fields. All data analysis was carried out by SPSS 22.0 software, each index was expressed by Means ± SD. Survival rates, including OS and DFS, were calculated using the Kaplan-Meier method and evaluated with the log-rank test. Cox proportional model was used to analyze the multivariate survival, and the independent risk factors affecting the survival time. Qualitative variables were compared using χ2 tests, and quantitative variables were compared using Wilcoxon tests (multi-group) or t test (two groups). Statistical significance was defined as p < 0.05.

## Results

### NLR ROC Curve and Changes in Different Groups

**Figure 1:**
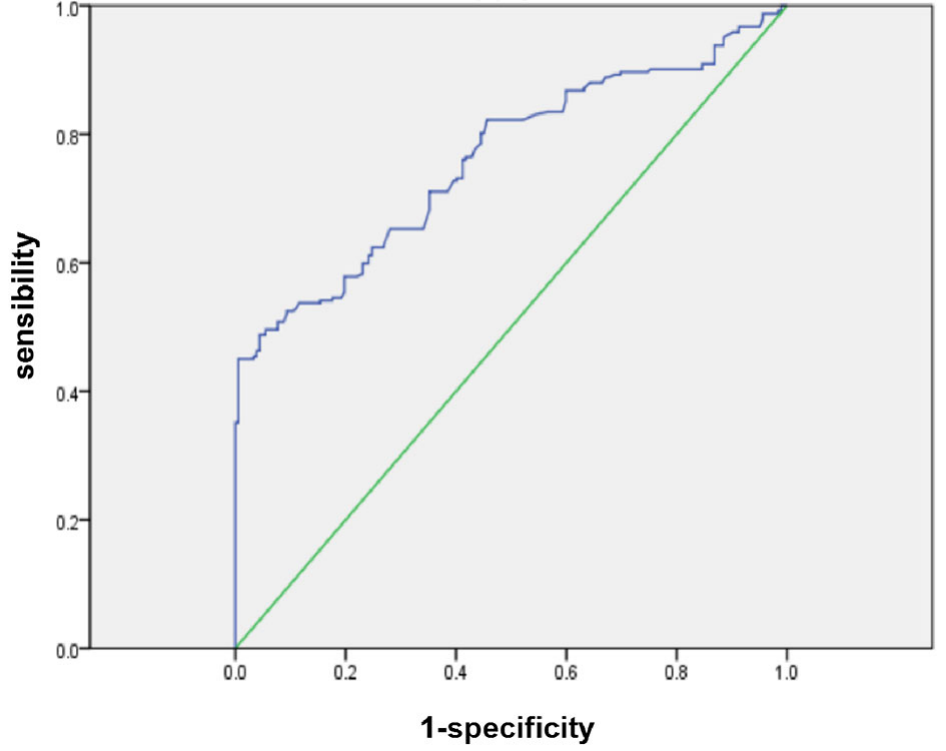
ROC curves for NLR in patients with PDAC (NLR, neutrophil-to-lymphocyte ratio; PDAC, pancreatic ductal adenocarcinoma).

According to the ROC curve, the optimal cutoff value of preoperative NLR that had a relatively high specificity was 2.9. The area under the ROC curves was 0.761 (P = 0.000) and 95% confidence interval (95% CI 0.716-0.805) (**Figure 1**). A cutoff value of 2.9 presented a sensitivity of 48.9% and a specificity of 95.6%.

The enrolled patients were divided into high NLR group and low NLR group according to the cutoff value. 118 patients (49%) identified as high NLR group had an elevated NLR (> 2.9), and 123 patients (51%) were identified as low NLR (≤ 2.9) group. There were significant differences among NLR with different degrees of differentiation (F = 2.826, P = 0.039), and also an obviously differences among neutrophil (NEUT) (F = 3.396, P = 0.019) but no differences among lymphocyte with different degrees of differentiation (F = 0.081, P = 0.462).

### The Preoperative NLR in Patients With PDAC and Its Relationship With Clinical Pathologic Characteristics

The 241 enrolled patients who underwent radical excision between January 2011and December 2019 consisted of 136 males and 105 females. Their mean age was 62.838 ± 10.742 years (yrs) with male 62.394 ± 10.550 yrs and female 63.409 ± 11.010 yrs. The date of operation was the starting point of follow-up and ended to May 2020. The longest follow-up time was 82 months, the shortest was 1 months, and the median follow-up time was 15 months. No patients were lost or withdraw during the study preformed.

**Figure 2:**
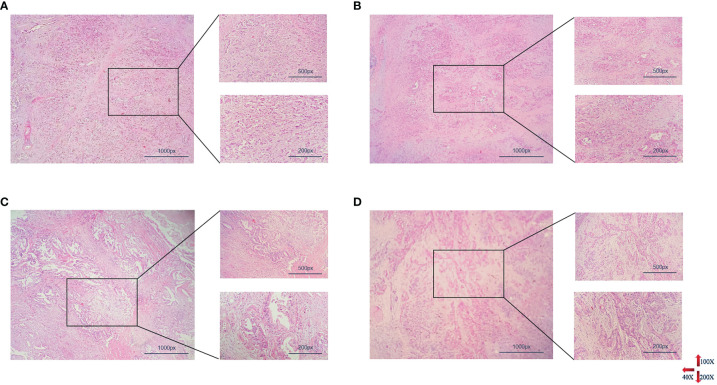
Histopathological results of PDAC with different degrees of differentiation. From **(A–D)** represented poorly differentiation, moderately-poorly differentiation, moderately differentiation, highly-moderately differentiation respectively (PDAC, pancreatic ductal adenocarcinoma).

Pathological analysis showed that all patients were PDAC with 31/241 of low differentiation, 56/241 of moderate-low differentiation, 126/241 of moderate differentiation, 28/241 of high-moderate or high differentiation (**Figure 2**). The average size of tumors was 3.779 ± 1.644 cm and 97/241 with vascular invaders, the pathological results of different groups are shown in **Table 1**. The relationship between preoperative peripheral blood NLR and clinical pathologic characteristics was investigated. As listed above (**Table 1**), 118 patients (49%) identified as high NLR group had an elevated NLR (> 2.9), and 123 patients (51%) were identified as low NLR (≤ 2.9) group. An elevated preoperative NLR level was closely correlated with the tumor size (range, > 4cm) (χ^2^ = 7.530; P=0.006), tumor differentiation (χ^2^ = 8.287; P = 0.040), clinical TNM stage(range, > II b) (χ^2 =^ 12.770; P=0.000), distant metastasis (χ^2^ = 7.858; P = 0.005), and bilirubin (TBIL *vs.*DBIL, *t* =-3.696 *vs.*-3.294, P = 0.000 *vs.*0.001). No obvious correlations with age, gender, CA-199, and other index (**Table 1**, P > 0.05).

**Table 1:** Background data preoperative and pathological results in various NLR groups.

Index	High NLR Group (n = 118)	Low NLR Group (n = 123)	*U*	*P*
Gender			2.719	0.099*^a^*
male	71	61		
female	47	62		
Age			3.751	0.053*^a^*
>55	100	85		
≤55	22	34		
Smoking (yes, %)	58 (49%)	51 (41%)	1.437	0.231*^a^*
Diabetes (yes, %)	50 (42%)	61 (50%)	1.264	0.261*^a^*
PBD (yes, %)	14 (12%)	9 (7%)	1.443	0.230*^a^*
NEUT	6.521 ± 3.079	3.129 ± 1.319	11.193	0.000*^b^*
Lymph	1.216 ± 0.820	1.804 ± 0.718	-5.925	0.000*^b^*
ALT (ng/ml)	(10-4583)	(10-336)	2.437	0.120*^c^*
TBIL	(3.6-552.8)	(5.5-275.4)	13.664	0.000*^c^*
DBIL	(2.1-505.5)	(0.79-219.5)	10.852	0.001*^c^*
Alb	37.079 ± 10.633	37.118 ± 4.715	-0.037	0.970*^b^*
CA199 (ng/ml)	42.89 (1.4-7000)	48.41 (2.6-7000)	1.324	0.251*^c^*
γ-GGT	6-1413	6-1957	1.055	0.305*^c^*
ALP	16-1398	47-1492	1.637	0.202*^c^*
Glu	7.308 ± 3.199	7.139 ± 2.708	0.439	0.661*^b^*
AMY	0.05-585	8-585	0.597	0.441*^c^*
Tumor site, n (%)			5.555	0.135*^b^*
uncinate process	79 (67%)	93 (76%)		
neck	6 (5%)	8 (7%)		
body and tail	33 (28%)	22 (18%)		
Tumor size, cm	4.057 ± 1.787	3.513 ± 1.543	2.597	0.010*^b^*
>4	45	27	7.530	0.006*^b^*
≤4	73	96		
Differentiation, n (%)			6.885	0.076*^a^*
poorly	21	10		
poorly-moderately	31	27		
moderately	54	68		
moderately-highly	12	18		
Vascular invasion, n (%)	45 (38%)	53 (43%)	8.874	0.096 *^a^*
LNs metastasis, n (%)	77 (65%)	81 (66%)	0.010	0.096 *^a^*
Nerve invasion, n (%)	108 (92%)	118 (96%)	0.010	0.096 *^a^*
Incisal Margin R0, n (%)	106 (90%)	121 (98%)	8.034	0.005*^b^*

^a^p-value from Chi-Squared Test or Fish’s exact test; ^b^p-value from Student’ t test; ^c^p-value from ANOVA; NLR, Neutrophil To Lymphocyte Ratio; PBD, preoperative biliary drainage; NEUT, neutrophil; ALT, Alanine transaminase; TBIL, total bilirubin; DBIL, direct bilirubin; Alb, albumin; CA19-9, carbohydrate antigen 199; γ-GGT, γ-gamma-glutamyl transpeptidase; ALP, A Lkaline Phosphatase; AMY, amylase; LN, lymph node.

### Diagnosis Value of NLR in PDAC Comparison With CA-199

Although there has lower correlation between NLR ≤ 2.90 and CA19-9 (r = 0.2193,95%CI 0.03943~0.3854; P = 0.408), but the NLR and CA19-9 was no correlation for all the patients (P = 0.408) and high NLR (NLR > 2.90) (P = 0.841). The diagnostic value of NLR to PDAC was analyzed by using the statistical diagnostic experimental method based on the currently recognized diagnostic standard of CA19-9. There has proved that NLR was with a sensitivity of 0.496 and a specificity of 0.515 in the diagnosis of PDAC (OR = 1.38, 95%CI of OR 0.94~2.02) as well as with a positive predictive value of 0.576 and a positive likelihood ratio of 1.022.

### Surgical Method and Vascular Anastomosis in Different NLR Group With PDAC

According to the results of preoperative imaging examination, 11 patients received palliative treatment (regarded as R1 resection), radical pancreaticoduodenectomy was performed in 160 cases, total pancreatectomy in 21 cases and distal pancreatectomy in 49 cases, of which 14 patients underwent R1 resection (2 patients in low NLR group, 1 patient in high NLR group) and the rest with R0 resection of a rate with 94.19%. There was no difference in the total number of lymph node dissection (19.789 ± 1.078, 19.297 ± 1.451, P = 0.785) and lymph node metastasis rate (2.252 ± 0.288, 3.297 ± 0.542, P = 0.087) between the two groups, nevertheless, there was significant difference in R0 resection rate (NLR ≤ 2.90 n = 2/123; NLR > 2.9 n = 12/118; χ^2^ = 8.034, P = 0.005). There was no significant difference in intraoperative blood loss (P = 0.699), blood transfusion (P = 0.753) and operation time (P = 0.687) between the two groups.

**Figure 3:**
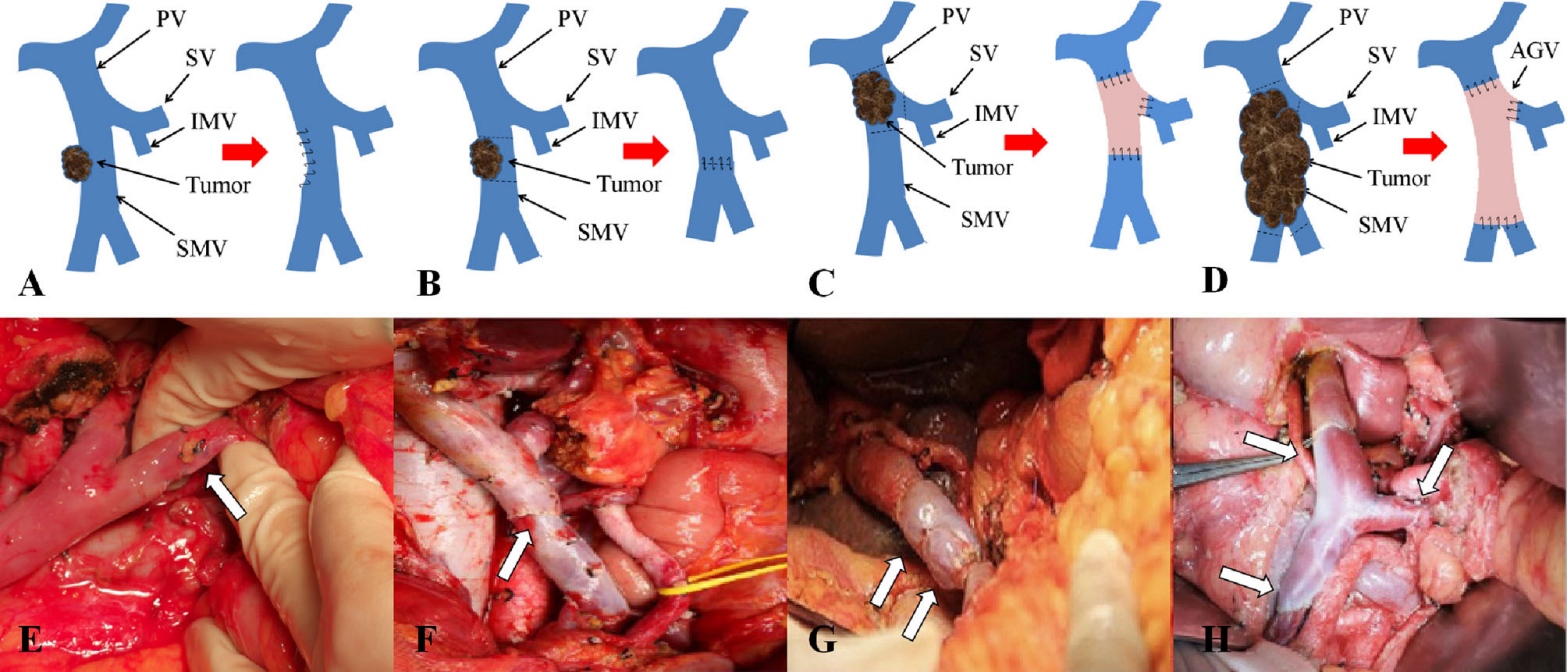
Chaoyang classification and management of venous invasion of borderline resectable pancreatic cancer. **(A, E)** Tumor invades superior mesenteric vein with wedge anastomosis; **(B, F)** Tumor invades superior mesenteric vein with end-to-end anastomosis; **(C, G)** Tumor invades portal vein with segmental vascular replacement; **(D, H)** Tumor invades the confluence of portal vein with branch vascular replacement.

Patients undergoing pancreaticoduodenectomy and total pancreatectomy were divided into two categories based on whether portal system invasion exists. Different vascular anastomosis and replacement methods were selected according to Chaoyang classification, which including partially excised and sutured directly (**Figures 3A, E**), end-to-end anastomosis (**Figures 3B, F**) and allogeneic vascular replacement with type I: segmental vascular replacement (**Figures 3C, G**), or type II: branch vascular replacement (**Figures 3D, H**).

These results supported the original hypothesis that comparing with palliative treatment, vascular resection or reconstruction was able to significantly improve the survival time of patients with vascular invasion (OS *vs.* DFS 9.909 *vs.* 7.727), different anastomosis or reconstruction methods could improve the OS and DFS remarkably, (P < 0.01) (**Table 2**) among which end-to-end anastomosis was the best (OS *vs.* DFS 30.154 *vs.* 27.192). As for vascular invasion, the OS and DFS of patients with vascular wall invasion less than 1/4 circumference diameter (Chaoyang type I) (P = 0.007) were significantly longer than those with invasion range greater than 1/4 circumference diameter (Chaoyang type II-IV) (P = 0.012) (**Table 2**).

**Table 2:** Univariate Analysis of risk factors for tumor recurrence and long-term survival in patients with PDAC.

Index	Variables (Number)	OS (Moths)			Index	Variables (Number)	DFS (Moths)		
		Mean	95%CI	*P value (U)*			Mean	95%CI	*P value (U)*
NLR	>2.9 (118)	16.030	12.149-19.912	0.000	NLR	>2.9 (118)	14.116	10.191-18.042	0.000
	≤2.9 (123)	36.574	30.763-42.385	67.869		≤2.9 (123)	34.196	27.989-40.402	61.696
Age	>55 (185)	24.247	20.038-28.456	0.032	Age	>55 (185)	21.087	17.058-26.105	0.039
	≤55 (56)	33.689	24.385-42.992	4.662		≤55 (56)	32.156	22.300-42.013	4.257
PBD	Yes(23)	15.957	9.268-22.627	0.044	PBD	Yes(23)	13.304	6.566-20.053	0.041
	No (218)	27.088	22.919-31.258	4.064		No (218)	25.104	20.622-29.547	4.179
CA-199	>37 (137)	21.735	17.426-26.044	0.038	CA-199	>37 (137)	19.041	14.585-23.497	0.031
	≤37 (104)	32.587	25.769-39.404	4.291		≤37 (104)	31.050	23.895-38.205	4.639
TNM	>II b (91)	14.368	11.332-17.405	0.000	TNM	>II b (91)	12.248	9.007-15.488	0.000
	≤II b (150)	32.431	27.075-37.787	26.988		≤II b (150)	30.427	24.740-36.113	24.185
Tumor Size	>4 (72)	19.284	13.882-24.687	0.002	Tumor Size	>4 (72)	17.648	12.080-23.216	0.003
	≤4 (169)	28.526	23.927-33.126	9.627		≤4 (169)	26.286	21.418-31.153	8.600
Tumor differentiation	I (31)	15.782	9.902-21.663	0.012	Tumor differentiation	I (31)	13.660	7.416-119.905	0.015
	II (56)	21.715	14.656-28.775			II (56)	20.455	13.172-27.738	
	III (126)	28.928	23.525-34.342	10.883		III (126)	26.658	20.918-32.397	10.552
	VI (28)	25.982	16.998-34.966			VI (28)	23.969	14.171-33.767	
Vascular anastomosis	I (11)	9.909	4.403-15.415	0.042	Vascular anastomosis	I (11)	7.727	2.206-13.249	0.056
	II (146)	26.997	21.998-31.996			II (146)	24.984	19.658-30.310	
	III (26)	30.154	17.696-42.616	8.224		III (26)	27.192	14.788-39.596	7.549
	IV(58)	22.212	16.444-27.979			IV(58)	19.932	13.734-26.131	
Operation Methods	I (11)	9.909	4.403-15.415	0.001	Operation Methods	I (11)	7.727	2.206-13.249	0.004
	II (160)	28.484	23.566-33.402			II (160)	26.461	21.241-31.681	
	III (21)	11.333	8.608-14.059	17.073		III (21)	9.122	6.601-11.643	13.563
	IV (49)	26.952	18.133-35.772			IV (49)	25.142	15.821-34.463	
Incisal Margin	R0 (227)	27.153	23.111-31.195	0.000	Incisal Margin	R0 (227)	25.155	20.853-29.457	0.000
	R1(14)	8.929	4.460-13.397	14.909		R1(14)	6.857	2.395-11.319	12.684
Lymphatic metastasis	Negative (83)	37.618	29.653-45.582	0.000	Lymphatic metastasis	Negative (83)	36.558	28.178-44.937	0.000
	Positive (158)	19.878	16.454-23.301	14.786		Positive (158)	16.993	13.540-20.446	15.328
Vascular invasion	I (11)	9.909	4.403-15.415	0.007	Vascular invasion	I (11)	7.727	2.206-13.249	0.012
	II (146)	28.543	23.386-33.700	9.966		II (146)	26.260	20.744-31.745	8.852
	III (84)	21.510	16.696-26.325			III (84)	19.644	14.494-24.794	

NLR, Neutrophil To Lymphocyte Ratio; PBD, preoperative biliary drainage; Tumor differentiation I: poorly, II: poorly-moderately, III: moderately, VI: moderately-highly; Vascular anastomosis I: palliative operation, II: partially excised and sutured directly, III end-to-end anastomosis, VI allogeneic vascular replacement; Operation Methods I: palliative operation, II: radical pancreaticoduodenectomy, III: total pancreatectomy, VI: distal pancreatectomy; Vascular invasion I: palliative operation, II: Chaoyang type I, III: Beyond Chaoyang type I.

### Preoperative NLR or Clinic-Pathologic Factors Associated With Postoperative DFS and OS

**Figure 4:**
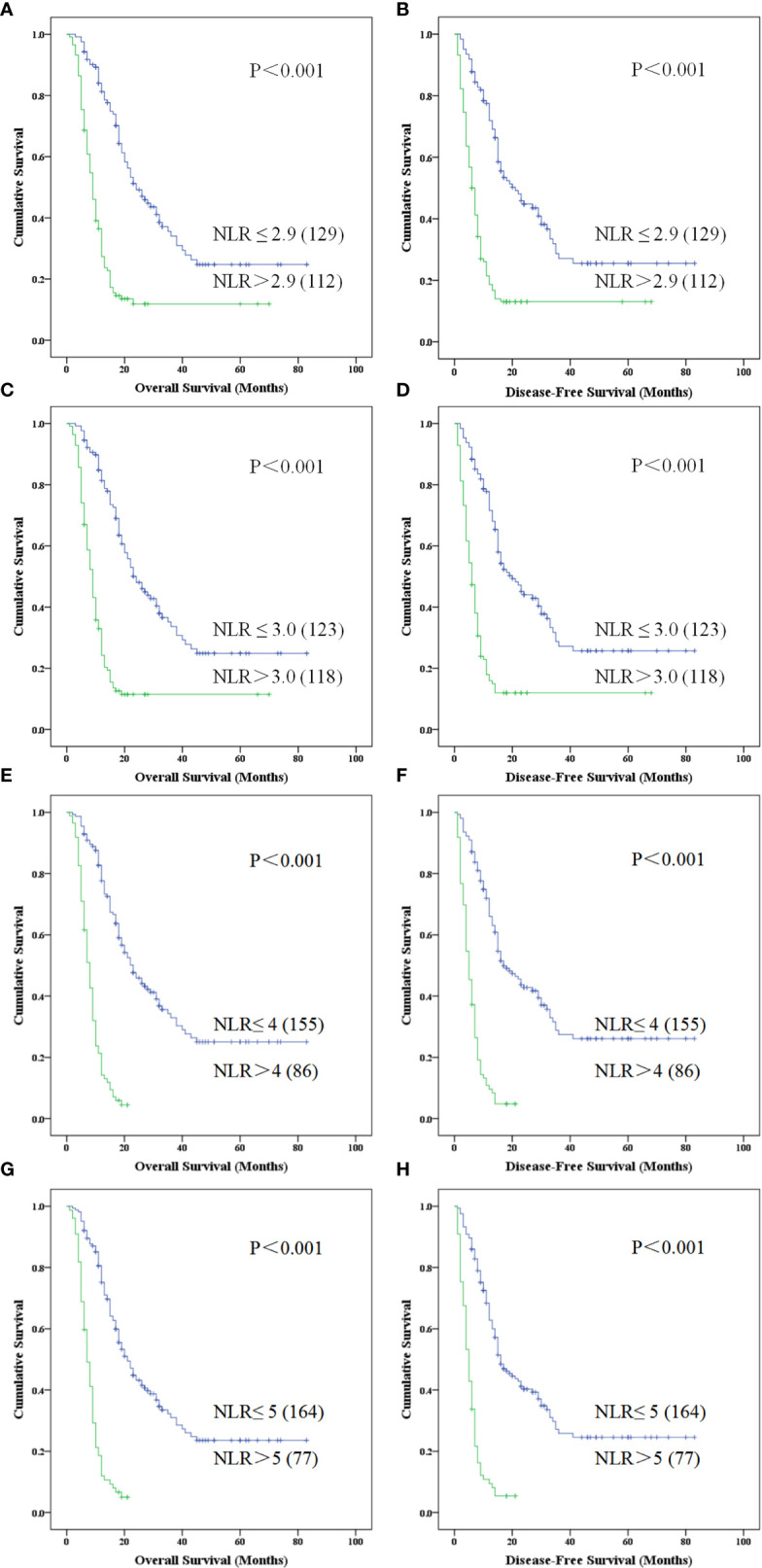
Kaplan-Meier curve for overall survival and disease free survival of patients with advanced PDCA by high NLR *vs*. low NLR for different NLR divided standards. **(A–H)** represented patients with higher NLR is associated with poorer survival which was obviously in NLR > 5 (P < 0.001) (NLR, neutrophil-to-lymphocyte ratio; PDAC, pancreatic ductal adenocarcinoma).

Kaplan-Meier survival analysis suggested that the OS (**Figure 4A**) and DFS (**Figure 4B**) of patients with NLR greater than 2.9 were shorter (all P < 0.001). Univariate analysis revealed that, clinical parameters of age, preoperative biliary drainage (PBD), CA19-9, TNM stage, tumor size, tumor differentiation, vascular anastomosis method, mode of operation, positive rate of incisal margin, lymph node metastasis, vascular invasion were all obvious associated both with DFS and OS (**Table 2**), however, gender, preoperative TB, DB, ALT, γGGT, ALP, Glu, amylase, history of smoking, history of diabetes and tumor location were not significantly correlated with OS and DFS (P > 0.05). Patients mean OS with NLR ≤ 2.90 and NLR > 2.9 was 36.574 (95% CI, 30.763-42.385) and 16.030 (95% CI, 12.149-19.912) months respectively (P < 0.001). Patients mean DFS with NLR ≤ 2.90 and NLR > 2.9 was 34.196 (95% CI, 27.989-40.402) and 14.116 (95% CI, 10.191-18.042) months respectively (P < 0.001). Moreover, the Age > 55, PBD, CA-199 > 37,TNM > II b, tumor size > 4cm, poorly differentiation, lymph node metastasis, incisal margin R1, vascular invasion were all associated with shorter OS and DFS (**Table 2**). Compared with palliative treatment, vascular resection/replacement could significantly improve the OS of patients, and there was significant difference among groups (P = 0.042), however, there was no significant difference in DFS among groups, which may be related to the short follow-up time.

As reported in previous literature, the cutoff value of NLR was selected as 3.0-5.0 (17, 18, 24–29) in different publications, so we also evaluated the patients with PDAC in this study using these cutoff values. Kaplan-Meier survival analysis showed that NLR > 3.0 (**Figures 4C, D**), 4.0 (**Figures 4E, F**) and 5.0 (**Figures 4G, H**) were associated with a relative shorter DFS and OS, but there are 86 (35.68%) cases with NLR > 4.0 (**Figures 4E, F**) and 77 (31.95%) cases with NLR > 5.0 (**Figures 4G, H**) in 241 patients with PDAC.

### Comparison of Pathology Differentiation in NLR Group Between Postoperative DFS and OS

On the basis of the above results, we performed survival analysis on the patients in different groups of NLR with different pathological differentiation. Kaplan-Meier survival analysis suggested that PDAC patients of low, moderate-low, moderate, moderate-high and high differentiation, with an elevated NLR > 2.9 displayed a shorter OS (χ^2^ = 8.718,15.291, 23.530, 61.760; P = 0.003, 0.000, 0.000, 0.000) and DFS (χ^2^ = 8.992, 14.012, 20.640, 16.389; P = 0.003, 0.000, 0.000, 0.000) when compared with NLR ≤ 2.90. Meanwhile, we performed the survival analysis in grouped of NLR cutoff was 3,4,5 which all shown the lower survival benefits both in OS and DFS(P < 0.001).

### Independent Predictors of DFS and OS in the Step Forward Multivariate Cox Proportional Hazards Model

In the presented study, the Cox proportional hazards model was used to evaluate the association between clinic and pathologic factors, surgical method and DFS/OS after surgical resection (**Table 3**). In addition to the correlation between vascular anastomosis method and OS, there remains six associated factors which including high NLR, resection margin R1, lymphatic metastasis, age > 55years, TNM stage of III-IV and tumor size > 4cm, were analyzed for OS and DFS by applying the step forward (condition LR) multivariate Cox proportional hazards model. The hazard ratio (HR), 95%CI, and P values concluded by Cox proportional hazards as listed in **Table 3**, high NLR, resection margin R1 and lymphatic metastasis were the most obviously independent risk predictor for OS and DFS with the HR > 2, meanwhile, beyond 55 years old, at TNM stage of III-IV or Tumor size > 4cm which were also the obvious independent risk predictor for OS and DFS with the HR ≤ 2 (**Table 3**).

**Table 3:** Cox multivariate proportional hazards of independent predictors on DFS and OS.

Variable	HR	95% CI	*P* Value
OS			
NLR (≤2.9 *vs.*>2.9)	3.138	2.234-4.410	0.000
Incisal margin (R0 *vs.*R1)	2.417	1.314-4.444	0.005
Lymphatic metastasis (yes *vs.*no)	2.019	1.427-2.858	0.000
Age,years (≤55 *vs.*>55)	1.611	1.089-2.385	0.017
TNM stage (I-II *vs.*III-IV)	1.506	1.087-2.087	0.014
Tumor size,cm (≤4 *vs.*>4)	1.441	1.025-2.026	0.035
DFS			
NLR (≤2.9 *vs.*>2.9)	2.970	2.121-4.158	0.000
Incisal margin (R0 *vs.*R1)	2.232	1.216-4.095	0.010
Lymphatic metastasis (yes *vs.*no)	2.072	1.463-2933	0.000
Age,years (≤55 *vs.*>55)	1.598	1.080-2.363	0.019
TNM stage (I-II *vs.*III-IV)	1.443	1.045-1.994	0.026
Tumor size,cm (≤4 *vs.*>4)	1.438	1.024-2.019	0.036

NLR, Neutrophil To Lymphocyte Ratio.

### Grouped Kaplan-Meier Analysis of DFS and OS by Risk Scores of PDAC Patients Based on Multivariate Cox Proportional Hazards Model

**Figure 5:**
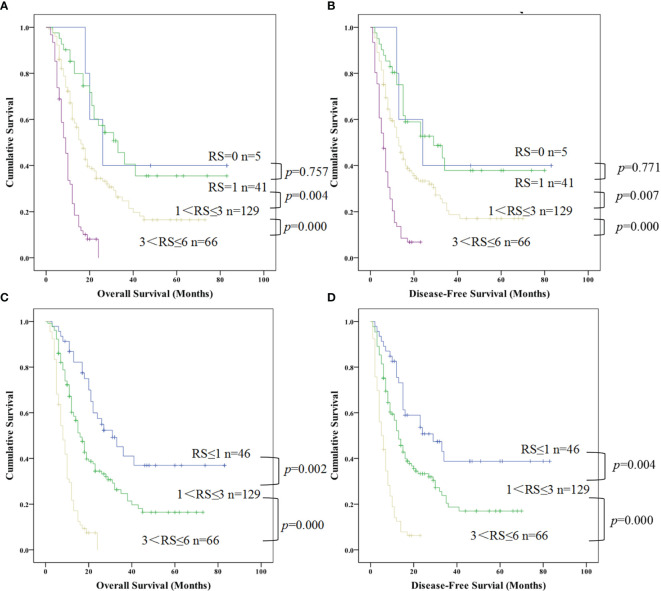
Kaplan-Meier curve for overall survival and disease free survival of patients with advanced PDCA by risk scores (RS) based on multivariate cox proportional hazards model. **(A, B)** represented the survival difference from the low RS to high RS; **(C, D)** represented the survival difference in the re-grouped RS groups which combined RS=0 and RS=1 as a new group (PDAC, pancreatic ductal adenocarcinoma; RS, risk scores).

Based on the above multivariate factor analysis results, we propose to establish a complex prognostic score calculating model by assigning value of multi-independent predictors **(**NLR, incisal margin, lymphatic metastasis, age, TNM stage, and tumor size). Each risk factor was allotted a score of 1 which all patients were grouped from risk scores (RS) 0 to 6 (RS = 0 reference without no any above factors as the control, and RS=6 reference with all of the above factors). Kaplan-Meier analysis of OS and DFS of the seven groups all indicated a significant survival difference (OS, χ^2^ = 149.247, P = 0.000; DFS, χ^2^ = 145.985, P = 0.000). There were only 5 cases in RS=0 group, and there was no difference in survival time between RS = 1 group and RS=0 group. In addition, the survival time of RS = 4, 5, 6 group were relatively short which were no more than two years (**Figures 5A, B**). Therefore, it was re-grouped into four groups: RS ≤ 1 (n = 46), = 2 (n = 74), = 3 (n = 55) and > 3 (n = 66). Survival analysis indicated that the survival time of RS ≤ 1, = 2 were more than 5 years, nevertheless, with the increase of RS factors, the survival time was gradually shortened (RS = 3). Once the RS > 3, patients with PDAC accounted for 27.38% of the total cases, meanwhile the survival time shortened rapidly, suggesting that the worse the prognosis (**Figures 5C, D**).

Moreover, we further combined RS=2 and 3 according to the results in Figures A, B above and regrouped patients with PDAC into four categories by their risk scores with RS=0, RS=1, 1 < RS ≤ 3, 3 < RS ≤ 6 which referred risk factors ranged from nonexistent to multiple effects on survival. Kaplan-Meier analysis also shown an obvious difference between different groups for OS and DFS (OS, χ2 = 74.051,P=0.000; DFS, χ2 = 68.721, P=0.000), but no difference between RS=1 and RS=0 both for OS and DFS (**Figures 5A, B**; P = 0.757 and P =0.771, respectively). Therefore, we combined these two groups as a new RS ≤ 1 group, redo survival analysis between the RS ≤ 1, 1 < RS ≤ 3, 3 < RS ≤ 6 respectively. That demonstrated that a distinguishable difference of OS (**Figure 5C**; RS ≤ 1 *vs*. 1 < RS ≤ 3, P = 0.002 and 1 < RS ≤ 3 *vs*. 3 < RS ≤ 6, P = 0.000) and DFS (**Figure 5D**; RS ≤ 1 *vs*. 1 < RS ≤ 3, P = 0.004 and 1 < RS ≤ 3 *vs*. 3 < RS ≤ 6, P = 0.000).

Surprisingly, the proportion of patients with PDAC with 3 < RS ≤ 6 was very high, occupying 27.38% (66/241) of total patients (**Figure 5**). The DFS and OS in the 66 patients with a score of 3 < RS ≤ 6 decreased sharply, and all these patients showed much shorter DFS and OS.

## Discussion

Pancreatic ductal adenocarcinoma (PDAC) accounts for more than 90% in pancreatic cancer, which is one of the most aggressive and lethal malignancies (1, 4, 5). Difficulties in early detection and diagnosis as well as R0 radical resection of PDAC contribute to the poor prognosis and high relapse to a great extent especially for advanced PDAC (3). Except CA19-9 as a marker for the diagnosis of pancreatic cancer, current studies on NLR in early pancreatic prognosis and diagnosis are deepening. Most studies believe that NLR can predict the prognosis of early pancreatic cancer, but there are few studies on advanced pancreatic cancer (17–20). Therefore, the enrolled PDAC patients of this study all met the criteria for Chaoyang classification of pancreatic cancer established by our center. In this presented study, evaluation of the prognostic predictive value of portal venous system resection and reconstruction in patients accepted radical surgical resection.

As pancreatectomy is considered the main treatment currently, all the enrolled patients were accepted curative excision with vascular resection and/or reconstruction according to the Chaoyang classification. The results showed improved survival benefits postoperative comparison with the palliative care patients with most obvious in those who have undergone pancreaticoduodenectomy. According to the Chaoyang classification and the actual intraoperative situation, the survival time of patients with the portal vein invasion was significantly prolonged after the resection of the vascular and end to end anastomosis. In addition to vascular invasion, lymph node metastasis was reported in 158 patients. All lymph nodes were biopsied intraoperatively until negative, but unfortunately, there were still 3 patients with positive lymph node biopsies.

Previous literature (30) asserted that vascular resection and reconstruction during the radical resection could improve the R0 resection rate and long-term survival. The R0 resection rate and survival time in different centers were various. Among them, the mean R0 resection rate was 71.4%, ranged from 37% in England to 87% in Germany. Meanwhile, the mean median survival time was 15.4 months, ranged from 14 months in America to 17 months in Japan. In this presented study, it displayed a R0 resection rate of 94.17% and a median survival time of 15 months. This results may due to the regulation of vascular resection for different types of invasion and the application of radical vascular replacement technique by Chaoyang classification of pancreatic cancers. We may consider that a radical surgery combined with vascular reconstruction has an obvious improvement in the prognosis and R0 resection rate of patients with advanced pancreatic cancer.

Further, single-factor survival curve analysis suggested that surgical approach, vascular anastomosis, vascular invasion, lymph node metastasis, and R0 resection were all related to survival benefit of OS and DFS. We consider that radical resection and anastomosis, including biopsy of all lymph nodes for advanced PDAC with vascular invasion is clinically beneficial and recommended.

NLR derived from the ratio of neutrophils to lymphocyte which both from white blood cells with important role in inflammatory response and tumor immunity. Neutrophils promote angiogenesis, tumorigenesis, metastasis, and tumor cell proliferation and survival and can also protect tumor cells from immune mediated destruction (31–33) which may through recruit regulatory T-cells into tumors *via* secretion of CCL17 (32). As we known, the immune response of hosts to tumor is lymphocyte-dependent. High elevated NLR patients usually with a relative lymphocytopenia, this may lead to a worse lymphocyte-mediated immune response to tumor, resulting in a shorter survival and the high risk tumor relapse and metastases (34).

Based on the literature regarding NLR, the purpose was aimed to evaluate the potential value of NLR as a prognostic indicator in patients with PDAC undergoing vascular reconstructive, so as to establish a simple computational model to predict tumor prognosis by combining NLR and biomarkers of oncological characteristics.

Studies have suggested that systemic inflammation is an important factor which can affect the progression and long-term survival of cancer patients (35). NLR is a simple parameter easily obtained to reflect a systemic immune inflammatory response elicited by the tumor (36). Despite research on NLR has been reported more with different methods in different populations, there is no general value at present. Forget et al. have identified a normal NLR values of 1.65 range from 0.78 to 3.53 in an adult, non-geriatric, population in good health (37), so the NLR cutoff value of 2.9 identified by ROC curve in our study was considered credible.

Current literature is conflicting regarding the prognostic value of NLR, with some showing a prognostic significance, and others demonstrating no significance on survival (19, 20). From our study, it first found that NLR was clearly related to the pathological differentiation of PDAC, secondly, an elevated NLR and NEUT was significantly showed in low differentiation patients, but no changes of lymphocytes. The results presented here suggest that NLR > 2.9 is highly associated with a worse survival benefits for PDAC. Meanwhile, it also represents a relative specificity value in PDAC diagnosis when carried out diagnosis experiments comparison with CA19-9. Some studies asserted that the diagnostic role of NLR is distinct from that of CA19-9 because of high NLR expression was not associated with CA19-9 levels (24). The correlation concluded in this study was the same as the previous, but due to the existing experimental results, We believe that NLR may also play a credible role in the diagnosis of advanced pancreatic cancer, especially in combination with CA19-9. However, the diagnostic value of high NLR in patients with negative CA19-9 indicators still needs to be verified in a large sample.

These results demonstrated that high NLR has a worse survival for advanced PDAC after curative excision with vascular resection and (or) reconstruction. The NLR > 2.9 was identified as a risk factor for lower survival in patients with PDAC. Patients with high elevated NLR (> 2.9) showed a significantly shorter OS and DFS than those with low NLR (≤ 2.9). With no clearly defined cutpoint of NLR, a cutoff value ranging from 2 to 5 has been widely used to define high/low NLR, of which 5 is the most widely used (16–18, 25–29, 38), therefore, we chose to perform a continuous analysis from NLR value of 3 to 5 for the OS and DFS. There showed a rather lower survival rate and shorter time as the NLR cutoff value increases gradually, with a survival no longer exceed 24 months of patients with NLR=4 or 5. These results were all consistent with the above literature reports (16–18, 25–29, 38), NLR > 3.0, 4.0 and 5.0 were also showed a shorter OS and DFS, but there were 86 (35.68%) cases and 77 (31.95%) cases with NLR > 4.0 and 5.0 in 241 patients with PDAC respectively comparison with 118 (48.96%) cases and 112 (46.47%) cases with NLR > 2.9 and 3.0 in 241 patients with PDAC respectively. That may mean a higher NLR exclude more advanced PADC patients and a cutoff value of 2.9 shows a higher sensitivity in diagnosis. Therefore, we considered that preoperative NLR of 2.9 is worthy as an optimal index with PDAC in this presented study, but also for other prospective clinical trials. However, the diagnostic value of high NLR in patients with negative CA19-9 indicators still needs to be verified in a large sample.

High elevated NLR and poor prognosis regarding PDAC was studied rather clearly, but the trend of NLR changes in different cancers and their effects on tumor immunity need to be elucidated. Furthermore, tumor-infiltrating lymphocytes (and specifically T cells) are responsible for mounting the antitumor response within the microenvironment (39) which reflected a weaker lymphocytic infiltration in tumor may with worse prognosis (34). Notably, PDAC has proven to have a unique and complex immune dysfunction with immunosuppressive cell types, tumor-supportive immune cells and defective inflammatory cells (40). Therefore, the damage mechanism to host immunity of the changes of neutrophil and T cell subsets and that infiltrated in tumor tissue are the next research direction.

In addition to NLR, surgical methods and vascular anastomosis, we also found that age (> 55), PBD, CA19-9 (> 37ng/ml), tumor size (> 4cm), TNM stage (III-IV), tumor differentiation (poorly or poorly-moderate differentiated) were obviously related with shorter OS and DFS by univariate survival analysis. These are all consistent with several previous reports that tumor size, TMN, CA19-9 were significant risk factor of recurrence after radical resection (41–44). There are also studies indicating that CA19-9 is an independent prognostic factor in PDAC (44–46). Although univariate analysis in this study showed that operation methods, vascular anastomosis, vascular invasion, tumor differentiation, CA-199 and preoperative biliary drainage (PBD) were preoperative prognostic predictors of poor DFS and OS, none of these factors were identified as independent predictors by multivariate analysis. This did not indicate that these factors are not associated with recurrence and metastasis and are not potential prognostic factors for advanced PDAC after curative resection.

Taken together, vascular reconstruction in radical resection of advanced PDAC displayed a longer survival benefits, but not an independent risk factor. What’s more, this study showed that high NLR (NLR > 2.90) was an independent predictor for DFS and OS of advanced PDAC undergoing vascular reconstruction.

Based on the Cox multivariate analysis, the results demonstrated that NLR, age, TNM stage, tumor size, lymphatic metastasis, and resection margin were independent prognostic factors for OS and DFS of the advanced PDAC. So, we have established a simple computational model of risk score (RS) with the above prognostic multiple-factor. In the RS model. In Cox multivariate analysis, NLR was the major component in predicting the survival and prognosis. According to the six predictors in the RS model, advanced PADC patients marked from 0 to 6 were grouped four RS groups (RS=0, RS=1, 1<RS ≤ 3, 3<RS ≤ 6). No matter which grouping method, the survival difference between groups was significantly with a no more than 24 months of survival time in group 3<RS ≤ 6.

It is worth noting that due to the limitations of the retrospective nature of this study and the small sample size of a single center, further multi-center, larger prospective studies are needed to verify this finding.

## Conclusion

Vascular reconstructive in radical resection of advanced PDAC improve survival, a higher elevated NLR (>2.90) was a negative predictor of DFS and OS in those patients accompanying portal system invasion. This study suggested that NLR might be a novel prognostic biomarker in advanced PDAC after curative resection.

## Data Availability Statement

The original contributions presented in the study are included in the article/supplementary material. Further inquiries can be directed to the corresponding authors.

## Ethics Statement

This study was approved by the Ethical Committee of Beijing Chao-Yang Hospital. All patients provided full written informed consent; written informed consent was obtained in accordance with the Declaration of Helsinki of the World Medical Association (Ethics approval and consent to participate: No.2020-D.-309-2).

## Author Contributions

(I) Conception and design: LZ, JW. (II) Administrative support: RL, QH. (III) Provision of study materials or patients: LZ, QH. (IV) Collection and assembly of data: JW, X-xZ and S-cL. (V) Data analysis and interpretation: LZ, L-cP and G-sD. All authors contributed to the article and approved the submitted version.

## Funding

This study was supported by: Beijing Municipal Science & Technology Commission, PR China (Grant No. Z181100001718164); Capital’s Funds for Health Improvement and Research, Beijing, PR China (CFH 2020-2-2036).

## Conflict of Interest

The authors declare that the research was conducted in the absence of any commercial or financial relationships that could be construed as a potential conflict of interest.

## Publisher’s Note

All claims expressed in this article are solely those of the authors and do not necessarily represent those of their affiliated organizations, or those of the publisher, the editors and the reviewers. Any product that may be evaluated in this article, or claim that may be made by its manufacturer, is not guaranteed or endorsed by the publisher.
